# Anthropometric, Nutritional, and Lifestyle Factors Involved in Predicting Food Addiction: An Agnostic Machine Learning Approach

**DOI:** 10.3390/diseases13080236

**Published:** 2025-07-24

**Authors:** Alejandro Díaz-Soler, Cristina Reche-García, Juan José Hernández-Morante

**Affiliations:** 1 Eating Disorders Research Unit, Universidad Católica de Murcia, Guadalupe, 30107 Murcia, Spain; adiaz591@alu.ucam.edu (A.D.-S.); jjhernandez@ucam.edu (J.J.H.-M.); 2 Multidisciplinary Research Group on Health Psychology, Universidad Católica de Murcia, Guadalupe, 30107 Murcia, Spain; 3 Research Group on Obesity, Diabetes and Metabolism, Instituto Murciano de Investigación Biomédico, Hospital Universitario “Virgen de la Arrixaca”, Ctra. El Palmar, 30120 Murcia, Spain

**Keywords:** food addiction, dietary intake, lifestyle, machine learning, SHAP, anthropometry

## Abstract

Food addiction (FA) is an emerging psychiatric condition that presents behavioral and neurobiological similarities with other addictions, and its early identification is essential to prevent the development of more severe disorders. The aim of the present study was to determine the ability of anthropometric measures, eating habits, symptoms related to eating disorders (ED), and lifestyle features to predict the symptoms of food addiction. Methodology: A cross-sectional study was conducted in a sample of 702 university students (77.3% women; age: 22 ± 6 years). The Food Frequency Questionnaire (FFQ), the Yale Food Addiction Scale 2.0 (YFAS 2.0), the Eating Attitudes Test (EAT-26), anthropometric measurements, and a set of self-report questions on substance use, physical activity level, and other questions were administered. A total of 6.4% of participants presented symptoms compatible with food addiction, and 8.1% were at risk for ED. Additionally, 26.5% reported daily smoking, 70.6% consumed alcohol, 2.9% used illicit drugs, and 29.4% took medication; 35.3% did not engage in physical activity. Individuals with food addiction had higher BMI (*p* = 0.010), waist circumference (*p* = 0.001), and body fat (*p* < 0.001) values, and a higher risk of eating disorders (*p* = 0.010) compared to those without this condition. In the multivariate logistic model, non-dairy beverage consumption (such as coffee or alcohol), vitamin D deficiency, and waist circumference predicted food addiction symptoms (R^2^_Nagelkerke_ = 0.349). Indeed, the machine learning approaches confirmed the influence of these variables. Conclusions: The prediction models allowed an accurate prediction of FA in the university students; moreover, the individualized approach improved the identification of people with FA, involving complex dimensions of eating behavior, body composition, and potential nutritional deficits not previously studied.

## 1. Introduction

Eating disorders (EDs) are serious mental disorders that involve dysfunctional eating patterns, distortions in body perception, and difficulties in emotional regulation. They are associated with high morbidity, an increased risk of mortality, and a high rate of years lived with disability among psychiatric diseases due to both their physical consequences and the mental health problems they entail [1]. These disorders usually appear during adolescence or early adulthood—a particularly vulnerable stage associated with neurodevelopment at this stage [2].

Currently, emerging subtypes of EDs have been described that may have the same or even greater prevalence and clinical relevance than the so-called classic ones (anorexia, bulimia, and binge eating) [3]. Among them, orthorexia, diabulimia, and the most studied of all, food addiction, stand out (which is not yet included in the DSM-5).

Food addiction (FA) is a clinical construct that describes a pattern of compulsive food consumption, especially in ultra-processed and highly palatable foods (high in fat and sugar). These foods activate brain reward circuits similar to those involved in substance addictions, with dopaminergic sensitization and conditioned reinforcement. From this perspective, FA emerges from the interaction between individual predisposing factors (genetic, epigenetic, or psychosocial) and continued exposure to foods with addictive potential [4]. People with FA have a dietary pattern characterized by a loss of control over food intake (which systematically exceeds the body’s needs), intense desire (craving), continued consumption despite negative consequences, and withdrawal symptoms when trying to restrict certain foods [5].

Food addiction has been associated with dysfunctions in the mesolimbic reward system, where exposure to high-calorie foods causes hyperactivity in the nucleus accumbens and orbitofrontal cortex, resulting in deficits in inhibitory control and behavioral self-regulation, similar to those observed in substance addiction and mediated by dysregulation of the frontostriatal circuit [6]. This dopaminergic circuit not only modulates preferences, desires, and gratification linked to food consumption but also plays a key role in preventing compulsive eating [7].

In specialized clinical settings, FA reaches very high prevalence rates, ranging between 70% and 90% in patients with bulimia nervosa or binge eating disorder (BED) [8], and about 32% in candidates for bariatric surgery [9]. However, in community samples, estimates are significantly lower and highly variable. In the general population, FA is present in a range of 16% to 20%, more frequently in adult women with overweight and obesity and ED clinical samples [10,11].

Among university students, an age group vulnerable to this type of disorder, FA prevalence rates have been observed at 6.7% in China [12] and 13% in Mexico [13]. They range from 6.4% [14] to 32% in Spain [15]. These data highlight the breadth and heterogeneity of the phenomenon at the cultural and contextual levels.

On the other hand, there is some controversy regarding FA and its association with anthropometric parameters. Although this disorder has traditionally been associated with obesity, recent studies have shown its presence in people of normal weight [16], and a relationship has been described between FA and dietary and lifestyle habits in university students [14].

In the Spanish university setting, lifestyle patterns are characterized by insufficient levels of physical activity, unbalanced diets, sleep disturbances, and substance use [17], along with stressors, such as residential mobility, academic pressure, and economic instability [18], which, as a whole, increase the risk of psychiatric disorders. Indeed, one study reported that 65.6% of university students are at risk of emotional disorders [19], while another study revealed that at least one-third of them suffered from one common mental disorder [20].

Given the growing concern about health habits among the university population, we want to understand the influence of these factors on food addiction to perform more appropriate and early detection. However, data analysis in psychology and psychiatry has been limited by the low statistical power of some studies and the lack of sufficiently advanced computational tools, which have restricted the ability to handle large volumes of data and build accurate predictive models [21]. However, recent advances in computational technologies have led to the integration of complex algorithms and machine learning methods combined with artificial intelligence-based techniques. This evolution has optimized the processing of highly complex data, improving both the efficiency and scalability of analyses [22]. As a result, the development of more robust predictive models has been strengthened, contributing to a more accurate characterization of clinical and psychopathological phenomena, as well as to more informed decision making in health and research contexts. They are used to minimize biases and preconceptions, discover novel or non-linear relationships, manage high-dimensional data, and leverage its predictive capacity.

In this context, the present study analyzes the presence of FA in university students with the aim of exploring predictors of food addiction using anthropometric, behavioral, and dietary variables and ML predictive modeling. In this work, we evaluated the prevalence of FA among university students and determined the association between FA and key anthropometric parameters, including body mass index (BMI), waist circumference, body fat percentage, and muscle mass; dietary parameters, including macro- and micronutrient intake; and lifestyle features, such as physical activity levels, symptoms related to eating disorders (EDs), use of tobacco, alcohol, and other psychoactive substances, in order to determine their ability to predict the symptomatology of FA.

The study hypothesis links anthropometric, dietary, and self-reported less healthy behaviors with an increased risk of FA diagnosis, which may help to predict FA.

## 2. Materials and Methods

### 2.1. Design and Participants

A cross-sectional study was designed to evaluate anthropometric measurements, eating habits, eating disorder symptoms, and lifestyle features in university students in order to predict food addiction (FA) symptoms.

The study participants were university students from the health sciences faculties at the Catholic University of Murcia (Spain) during the 2023/2024 academic year. Data were collected online, with no compensation provided for participation. Those students who voluntarily agreed to participate were included. Students who did not adequately complete the questionnaires and those with previously diagnosed eating disorders or health problems requiring chronic medication were excluded. From all the students, 720 Caucasian students participated in the study (557 women and 163 men, with a mean age of 22 ± 6 years).

The survey was conducted with prior written authorization from the Ethics Committee of the Catholic University of Murcia (Code: CE042008) and in compliance with the Declaration of Helsinki. Participants were informed about the study design, both orally and in writing. The research was also explained, including the objective, the need for confidentiality, and anonymity of the data.

### 2.2. Measures

#### 2.2.1. Demographics and Lifestyle Data

Information about these variables was collected using an ad hoc questionnaire specifically designed for this study. It included sociodemographic (sex, age) and behavioral variables, with questions about weekly frequency of physical activity, tobacco use (yes/no), alcohol use (yes/no), and medication use (yes/no). The presence of allergies, intolerances, dietary restrictions, or weight changes were also included; these questions were taken from the Clinical Evaluation Protocol of the Spanish Society of Clinical and Metabolic Nutrition [23].

#### 2.2.2. Food Frequency

In the present study, an online version of the Food Frequency Questionnaire (FFQ) was used to assess nutrient and food intake (https://bio-hpc.ucam.edu/ucamhealth/ last accessed on 23 July 2025).

This questionnaire is an updated version of the original FFQ, which collects information on 136 foods instead of the original 118, considering nine frequencies ranging from “never or almost never” to “more than six times a day”. The updated version incorporates foods widely consumed in Spain and was designed to adapt to the evolving Spanish diet [24].

#### 2.2.3. Eating Disorders Screening

The Eating Attitudes Test (EAT-26), developed in the late 1970s, is a widely used tool for the early detection of dysfunctional eating behaviors and attitudes associated with eating disorders (EDs) [25]; therefore, it was used in this work.

The EAT-26 is structured into three complementary sections that allow for a multidimensional assessment of eating patterns. The test includes 26 items that explore attitudes and behaviors related to eating, rated on a six-point Likert scale ranging from “always” to “never”. This design allows for the identification of both the presence of eating concerns and the manifestation of specific behaviors, offering a comprehensive view of the risk of eating disorders in the previous six months [26]. The reliability of the EAT-26 questionnaire in Spanish samples shows a Cronbach’s alpha of 0.93 [27].

#### 2.2.4. Food Addiction

The detection of FA was carried out with the Yale Food Addiction Scale 2.0 (YFAS 2.0) [28], a validated tool that assesses the presence and severity of food addiction based on the DSM-5 diagnostic criteria for substance use disorders. It consists of 35 items rated on an eight-point Likert scale (0 = never; 7 = every day), which identifies both the frequency of symptoms and the existence of clinically significant impairment or distress. A diagnosis of food addiction requires the presence of at least one symptom, along with evidence of clinical distress. Severity is classified as mild (2–3 symptoms), moderate (4–5 symptoms), or severe (6–11 symptoms), allowing for quantitative assessment of the behavioral and emotional impact linked to the dysfunctional consumption of highly palatable foods. The Spanish version has a Cronbach’s alpha of 0.94 [29].

#### 2.2.5. Anthropometric Variables

For anthropometric and body composition assessments, a TANITA MC-780^®^ multi-frequency analyzer (TANITA Corporation of America, INC., Arlington Heights, IL, USA) was used. Height was measured with a Harpenden model stadiometer according to the Frankfurt plane standards. Body mass index (BMI) was calculated using the formula weight (kg)/height^2^ (m^2^), with obesity being considered a BMI greater than 30 kg/m^2^. Waist circumference was measured three times above the iliac crests using an inextensible tape measure without compressing the skin. The percentage of body fat was obtained using electrical bioimpedance. All measurements were performed by a single operator with previous experience in anthropometric determinations in three non-consecutive repetitions.

### 2.3. Statistical Analysis

Frequency and other descriptive statistics were calculated for the sociodemographic variables and for the remaining predictor variables included in the analysis. Chi-square tests were used to examine the association between FA diagnosis and sex, as well as with other categorical variables. Differences in continuous variables, such as age, were assessed using Student’s *t*-test for independent samples. A multivariate logistic regression (LR) model was subsequently constructed to identify the factors associated with FA. The LR model was developed using a backward selection strategy, incorporating only those variables that showed a statistically significant influence in the bivariate analysis. Adjusted odds ratios (ORs) were calculated with their corresponding 95% confidence intervals.

To compare and optimize the results obtained in the regression model, two supervised machine learning algorithms, random forest (RF) and gradient boosting (GB), were implemented. Both methods allow the construction of non-linear models without requiring strict parametric assumptions. To do this, the dataset was divided into a training set (80%) and a validation set (20%), and cross-validation was conducted. In the RF model, multiple decision trees were randomly constructed from subsets of the training set. This approach is especially robust to overfitting and provides a direct estimate of the relative importance of each predictor variable, determined as an increase in node purity. The GB model is based on the sequential construction of decision trees, in which each tree attempts to correct the errors of the previous one. In both models, performance metrics, such as accuracy, sensitivity (recall), and specificity, were evaluated, comparing them with those obtained by the logistic regression model.

Finally, to individually interpret the prediction models, SHAP values were calculated. This methodology assigns each variable a quantifiable contribution to the prediction, thus allowing for the identification of which factors increase or decrease the probability of FA diagnosis in every individual. Global graphs and individual representations (barplots) were generated to visualize the relative influence of the variables for different patients. The dependent variable was binary (addiction vs. non-addiction), and the independent variables included sociodemographic, anthropometric, clinical, and dietary characteristics. The model was trained with the following hyperparameters: n_estimators = 100, learning_rate = 0.1, and max_depth = 4. The SHAP analysis was implemented using the TreeExplainer class optimized for tree-based models.

Data analyses were performed using SPSS software, version 27.0 (IBM SPSS Statistics, Armonk, NY, USA), and R software (R release, version 4.4.2). RF and GB models were developed with the libraries ‘randomForest’ and ‘xgboost’, respectively. Other libraries like ‘caret’ and ‘pROC’ were also used for metric evaluation. The SHAP interpretability analysis was developed in Python 3.12.4 using the ‘xgboost’ libraries for modeling, ‘scikit-learn’ for dataset validation and partitioning, and ‘shap’ for calculating and interpreting SHAP values.

## 3. Results

### 3.1. Baseline Characteristics of Participants

From the 702 participants who completed the study, most were women (557 women, 77.3%), with a mean age of 22 ± 6 years. The prevalence of food addiction (FA) was 6.4% (n = 46), with no statistically significant associations with sex (chi-square = 0.265; *p* = 0.607) or age (*t* = 1.883; *p* = 0.060). Among those with food addiction, half met the severity criteria, 31.1% had moderate symptoms, and 10.9% had light symptoms.

When analyzing the YFAS-specific symptoms, the most frequent was the “*inability to stop eating certain foods*”, reported by 23.5% of participants, followed by a “*willingness to use in physically dangerous situations*”, presented in 17.8% of the participants. On the other hand, the least reported symptom was “*tolerance*”, with only 4.3% prevalence.

Anthropometrically, the mean BMI placed individuals within the normal range. Half of the addicted students were of normal weight. A very weak significance was found between BMI and FA-related symptoms (*r* = 0.125). Individuals with FA showed higher weight, BMI, waist, and body fat percentage values compared to their counterparts, although no significant differences were observed in muscle mass, as reflected in parameters such as fat-free mass and arm muscle circumference. These results suggest an association between food addiction and greater adiposity, without compromising lean mass (Table 1).

Regarding lifestyle habits, 26.5% of people with FA reported smoking daily, 70.6% reported frequent alcohol consumption, 2.9% reported using illicit drugs, and 29.4% reported taking chronic medication. Thirty-five percent did not engage in any kind of physical activity. In addition, 14.7% had diagnosed nutritional allergies and/or intolerances, 50% had self-imposed dietetic restriction (not medical), and 41.2% had recent weight changes (Table 1).

### 3.2. Nutrient and Food Intake

The most important observation regarding dietary habits was the high similarity between people with and without FA. As shown in Table S1, there were no statistically significant differences regarding macro- or micronutrient intake, and only vitamin D intake was statistically significantly higher in those without FA (*p* = 0.035). When food intake was evaluated, significant differences regarding meat consumption and non-dairy drink consumption (such as coffee or alcohol) attending the FA diagnosis were observed (Table S1). Interestingly, sugary food consumption (including table sugar, chocolate snacks, marmalade, honey, etc.) was lower in those individuals with FA, but in this case, the differences were only marginally significant (*p* = 0.056).

### 3.3. Screening for Eating Disorders and Food Addiction

In the participants, the risk of developing an eating disorder exceeded that of food addiction (6.4%), with 8.1% of participants scoring above the cut-off point on the EAT-26 questionnaire, indicative of a possible eating disorder; statistically significant differences (*p* = 0.010) were observed in the EAT-26 score, with people with food addiction being likelier to also be at risk for eating disorders. Indeed, ED risk and FA were concomitant in 2.4% of the participants. Moreover, the EAT-26 score was positively associated with the symptoms of FA (*r* = 0.410, *p* < 0.001).

### 3.4. Models for the Prediction of Food Addiction

The prediction of FA was performed in three steps. First, a logistic regression analysis was conducted using food addiction diagnosis as the dependent variable. Second, two machine learning algorithms were employed to try to improve the precision of the logistic regression (LR model). Finally, an individualized agnostic machine learning model was developed to calculate SHAP values for every individual.

Thus, the dates derived from the LR model are shown in Table 2. Only those variables with a statistically significant difference in the bivariate analysis were included. In addition, those variables with VIF > 5 were also excluded from the model. Finally, all variables except body fat and diet restriction (self-imposed) significantly influenced the FA prediction.

**Figure 1:**
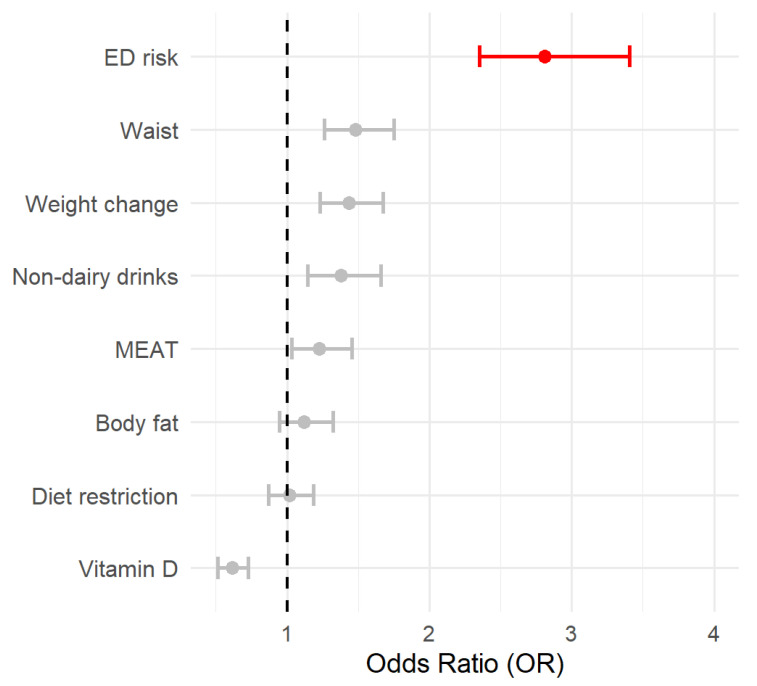
The forest plot represents the OR associated with the different variables included in the logistic model. OR > 2.5 is highlighted in red. The circle represents the OR value, and the lines are the 95% CI.

The accuracy, sensitivity, and specificity were 0.75, 0.74, and 0.90, respectively, which indicates good fitness. To further interpret the relevance of every factor, the OR and their respective CI95% were estimated and shown in Figure 1.

**Figure 2:**
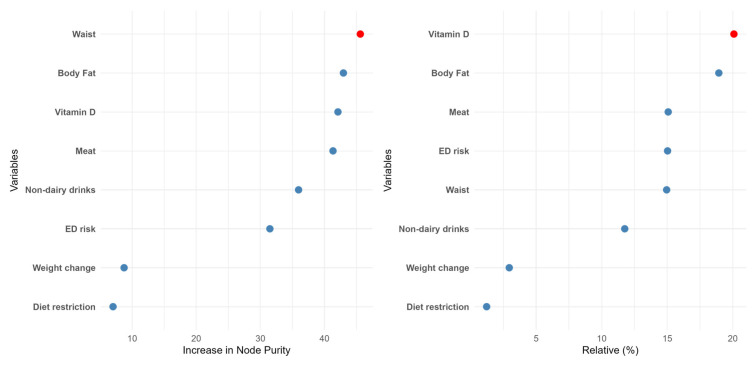
The relative importance of the different features according to the random forest (left panel) or the gradient boosting (right panel) algorithms.

Two different machine learning algorithms were developed in the present work: random forest (RF) and gradient boosting (GB). Attending to the RF data (Figure 2), waist was the most important feature in predicting food addiction, followed by body fat and Vitamin D intake. Interestingly, the ED risk, which was the most relevant factor in the LR model, dropped to the sixth position. The other ML procedure also yielded different results from the LR model. Concretely, in the GB data, the relative importance of the different features was in line with the previous model, although in this case, the intake of vitamin D, a “protective” factor, was the most relevant factor. Nevertheless, in both ML models, the relevance of ED risk was modest, and the less important determinants of food addiction diagnosis were self-imposed diet restriction and recent changes in body weight. The performance of these methods was also high in terms of accuracy and specificity (Figure S1).

**Figure 3:**
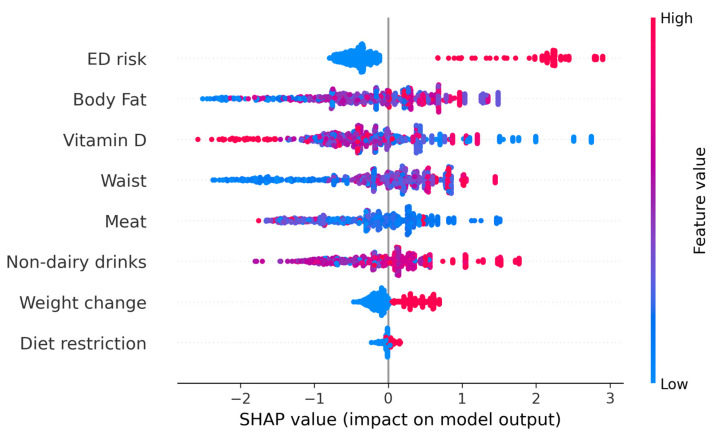

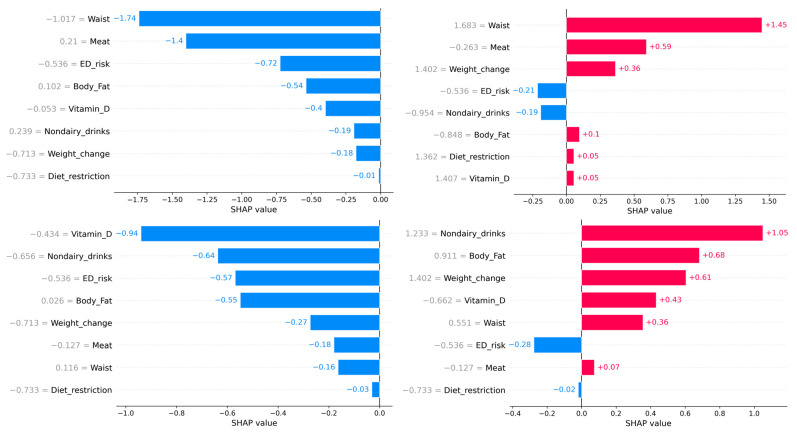
The upper figure represents the beeswarm plot of all individuals to show the importance of every feature. The lower barplots represent individual predictions of food addiction. The left panel indicates participants with a low probability of food addiction. The right lower panels indicate participants with a high probability of food addiction. Although the probability of all individuals was similar, the relative importance of every feature depended on the participant.

We further evaluated the possibility of determining individual risk of developing food addiction. To do this, SHapley Additive exPlanations (SHAP) values were determined to better interpret the results of the ML models. The SHAP values represent the contribution of that particular feature to the model’s prediction for any individual, compared to the average of the predictions. Figure 3 represents the beeswarm plot and individual barplots showing the global importance of the features in the model, as well as two examples of individuals with low probability of food addiction (f(x) = 0) and two with high probability of food addiction (f(x) = 1). The SHAP analysis indicates that ED risk was an important determinant: when a person was at risk for an ED, the probability of food addiction was very high. However, the reverse was not true, as being at low risk for an ED risk had little impact on predicting a low likelihood of food addiction. Overall, when a person has an EAT-26 score indicative of ED risk, they more likely to have FA; if they do not, other factors may be more relevant.

## 4. Discussion

This study determined the ability of anthropometric measures, eating habits, symptoms related to eating disorders (ED), and lifestyle factors to predict the symptoms of food addiction (FA). Through various prediction models, it was possible to determine the probability of a university student developing FA, which will be of great interest considering the high prevalence of this disorder in these environments.

A 6.4% prevalence rate of FA was found, which matches the percentage reported in a previous study also conducted in a Spanish university population [14]. In adolescents, this percentage was higher [16]. This may suggest that as individuals transition to adulthood, they may develop more protective factors because neural development is more advanced, which may help to establish a less problematic relationship with food.

No differences in FA were observed between men and women, as in previous research on Spanish university populations [15], but this differs from those found in North American university students [30]. It appears that FA is not a disorder with sex differences in our cultural context, contrary to what happens with classical EDs like anorexia or bulimia nervosa, which are characterized by a higher prevalence in women. Moreover, university students diagnosed with FA had a higher BMI and waist circumference, and they showed greater adiposity without compromising lean mass, a situation that could be a consequence of the differential dietary habits depending on the presence or absence of an FA diagnosis [16]. There was a weak relationship between BMI and FA-related symptoms in the participants. These findings are similar to the previous literature associating FA with obesity risk [31], although they differ from the lack of association recently found between FA and BMI in university students [15].

There was no difference in lifestyle habits between those diagnosed with food addiction and those not, such as sedentary lifestyles [14] or drug use [32]. However, there were differences in nutritional habits, weight changes, and self-imposed dietary restrictions among those with FA. This combination is concerning, as it may reflect a pattern of alternating between strict control and episodes of dysregulation. These people could be on compensatory diets that would at the same time facilitate their addiction.

The dietary profile of individuals with food addiction was expected to show a higher intake of total fat, protein, carbohydrates, sugar, and processed or energy-dense foods [16,33]. Moreover, higher amounts of certain micronutrients, such as sodium, calcium, omega-3, and omega-6 fatty acids, among others [34]. However, addicted college students had lower amounts of vitamin D and higher consumption of nondairy beverages (such as coffee, energy drinks, or alcohol), with no differences in energy intake and the rest of the micro- and macronutrients assessed. This indicates the peculiarities of this population of college students and suggests a new path of study.

Students with FA present with more symptoms related to eating disorders. It should be noted that among the most studied comorbid disorders in food addiction are eating disorders, such as binge eating, bulimia nervosa, and obesity [4].

In order to predict the presence or absence of FA, the multivariate analysis model identified three significant predictors of FA: non-dairy beverage consumption, vitamin D deficiency, and waist circumference. Together, these variables explained 22–35% of the variance in the FA diagnosis. This finding reinforces the idea that FA is not simply a matter of caloric intake or BMI but involves more complex dimensions of eating behavior, body composition, and potential nutritional deficiencies. Indeed, ML models also highlighted the role of body fat and vitamin D intake as predominant factors in predicting FA.

It is particularly revealing that Body Mass Index (BMI), often used as a reference in health studies, did not behave as a significant predictor. This finding reinforces the need to move beyond a view focused exclusively on body weight as the sole marker of nutritional or psychological well-being. As evidenced in this study, FA can manifest in the absence of obesity, highlighting the prominent behavioral and emotional nature of this mental condition [16].

The application of ML models in healthcare environments has increased hugely in recent years, yielding hopeful advances to improve patient diagnosis as well as other health resources [8,35]. The individual ML model based on SHAP values aimed to predict the likelihood of FA in university students. This model, which is easy to implement, will facilitate specific prevention interventions to avoid the development of FA. Previously, works like the one conducted by Cerasa et al. used ML approaches to predict ED through several biomarkers, but as the authors stated, findings should be considered preliminary given the small sample size investigated [36]. Arold et al. also employed ML techniques to predict anorexia nervosa long-term outcomes [37]. However, to the best of our knowledge, this is the first report to use ML in the context of FA.

While ML models are not without limitations, such as the need for a high amount of data or the lack of practical interpretability, the present work makes up for these deficiencies through two different approaches. On the one hand, an elevated sample size was evaluated, which improved the ML metrics, confirming the suitability of the current model. On the other hand, the calculation of individualized SHAP values allows an easy and accurate evaluation of the main features determining FA in each individual. While simple ML models provide a prediction (e.g., outcome risk = 75%), they cannot clarify how or why the outcome occurred. In contrast, SHAP values offer detailed case-by-case explanations, showing exactly how each variable (e.g., body fat, vitamin D, etc.) influences the prediction of whether FA-related symptoms will occur [38].

Some limitations of the study include the following: First, the questionnaires were self-reported, which can introduce bias, especially in the diagnosis of FA and ED, which could have been underestimated. Furthermore, the cross-sectional design does not allow us to determine whether these variables are simply related to the development of food addiction. Although our objective was to develop predictive models that have achieved excellent performance, prospective longitudinal studies would be necessary to determine whether these factors are responsible for addiction. From a practical perspective, these findings suggest that it would be advisable to design specific interventions for the university population, incorporating nutritional education, emotional regulation techniques and monitoring anthropometric markers such as waist circumference. These measures could act preventively and allow for the monitoring of clinical indicators.

Finally, we propose future longitudinal studies and the combined use of qualitative and quantitative methodologies to provide a deeper understanding of the patterns underlying FA and to confirm that both the LR and the ML models are adequate to predict FA in university settings. Furthermore, we propose to address possible unexplored gender differences in our predominantly female sample.

## 5. Conclusions

FA was present in 6.4% of the university students, with no differences by sex. The anthropometry of students with FA revealed larger BMI, waist circumference, and body fat percentage. There were no differences in the lifestyle variables evaluated, but there were differences in their eating habits, self-imposed calorie restrictions, and recent weight changes. This information allowed the development of several prediction models, from conventional techniques such as logistic regression (LR) to individualized prediction machine learning-based models. Interestingly, both LR and individualized predictions highlighted the relevance of ED symptomatology. This emphasized the need to develop patient-specific prediction models, since the regression model may be effective if the individual does have ED symptoms, but if they do not, the risk of FA may be underestimated. Thus, models like the present one could allow for a more precise assessment of the influence of other characteristics, such as body fat percentage or vitamin D intake.

In order to predict the presence of FA, it would be necessary to first detect the risk of an ED. Without ED symptomatology, other factors—such as non-dairy beverage consumption (like coffee, energy drinks, or alcohol), vitamin D deficiency, and waist circumference—predicted food addiction symptoms, reflecting more complex dimensions of eating behavior, body composition, lifestyle habits, and potential nutritional deficits not previously studied. In contrast, widely studied variables such as BMI, drug use, and frequency of physical activity did not explain symptoms related to food addiction, at least in the present university population.

## Figures and Tables

**Table 1:** Anthropometric factors, lifestyle factors, and nutritional factors involved in predicting food addiction.

	FA	NO FA	*p*
Normal weight (%)	50	73	<0.001 *
Overweight (%)	50	27	
Body Mass Index	25.90	23.34	0.010 *
Body fat (%)	31.44	26.48	0.001 *
Fat-Free Mass (kg)	48.60	47.34	0.429
Waist (cm)	85.8	78.7	0.001 *
AMC (mm)	230.89	234.83	0.629
Smoking (%)	26.5	19.8	0.346
Alcohol use (%)	70.6	60.2	0.229
Drug use (%)	2.9	1.4	0.475
Medication (%)	29.4	21.5	0.278
Physical activity (%)	64.7	68.5	0.646
Allergies/intolerances (%)	14.7	20.8	0.394
Dietetic restriction (%)	50	28.2	0.007 *
Weight changes (%)	41.2	25.2	0.0039 *

* Significant mean difference at the *p* < 0.05 level.

**Table 2:** Regression logistic analysis using food addiction diagnosis as the dependent variable and those variables with statistically significant differences in the bivariate analysis.

	B	SD B	Wald χ^2^	Z	*p*-Value	OR	95% CI OR
Waist	0.393	0.084	21.92	4.682	<0.001 **	1.48	1.26–1.75
Body fat	0.111	0.085	1.72	1.313	0.189	1.12	0.95–1.32
ED risk	1.034	0.094	120.72	10.987	<0.001 **	2.81	2.35–3.41
Weight change	0.361	0.079	20.79	4.560	<0.001 **	1.43	1.23–1.68
Diet restriction	0.016	0.078	0.04	0.201	0.840	1.02	0.87–1.18
Meat	0.206	0.087	5.55	2.356	0.018 **	1.23	1.03–1.46
Non-dairy drink	0.320	0.095	11.42	3.379	0.001 **	1.38	1.15–1.66
Vitamin D	−0.487	0.089	30.22	−5.497	<0.001 **	0.61	0.51–0.73

Weight and BMI were excluded for showing a VIF > 5. R^2^_McFadden_: 0.220; R^2^_Cox-Snell_: 0.262; R^2^_Nagelkerke_: 0.349; **: highly significant.

## Data Availability

The original contributions presented in this study are included in the article/Supplementary Materials. Further inquiries can be directed to the corresponding author.

## Supplementary Materials

The following supporting information can be downloaded at: https://www.mdpi.com/article/10.3390/diseases13080236/s1. Figure S1: ROC curves of the logistic regression, the random forest, and the gradient boosting models; Table S1: Nutrient and food group information based on FA diagnosis.

## Abbreviations

The following abbreviations are used in this manuscript:

BMI	Body Mass Index
EAT-26	Eating Attitudes Test-26
ED	Eating Disorder
FA	Food Addiction
FFQ	Food Frequency Questionnaire
SHAP	Shapley Additive Explanations
YFAS 2.0	Yale Food Addiction Scale 2.0
