# Supplementary material for: Anthropometric, Nutritional, and Lifestyle Factors Involved in Predicting Food Addiction: An Agnostic Machine Learning Approach
Source: Diseases. 2025 Jul 24;13(8):236. doi: 10.3390/diseases13080236 (PMC12385177; doi:10.3390/diseases13080236)
Supplement: Supplementary file 1 [file diseases-13-00236-s001.zip › diseases-3761071-supplementary.pdf]

SUPPLEMENTARY MATERIAL

Figure S1. ROC curves of the logistic regression, the random forest and the gradient boosting models.

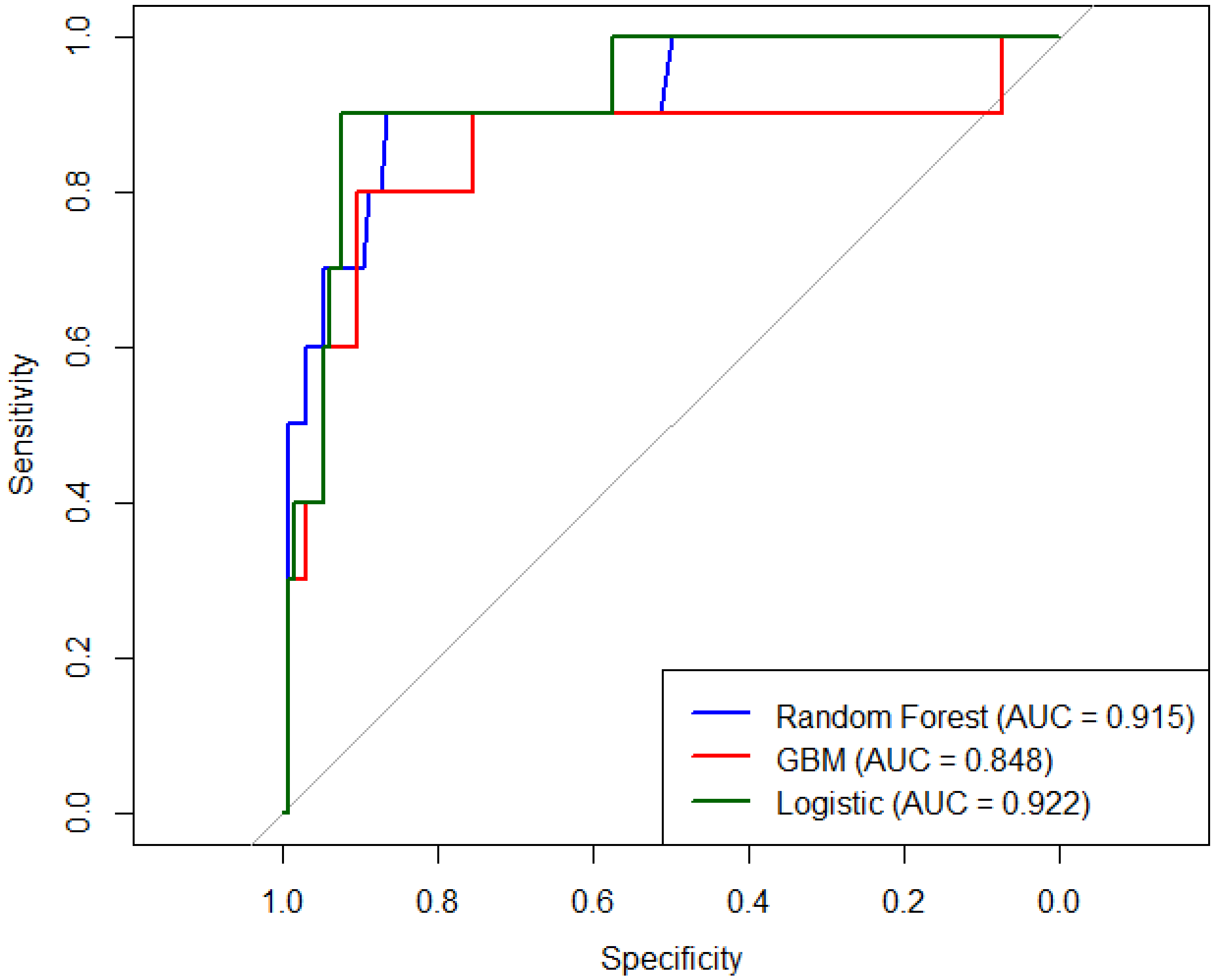

Table S1. Nutrient and food group information based in FA diagnosis

	**Food Addiction**	**No Food addiction**	***p*-value**
Macronutrients			
Kcal/day	2761 ± 827	2882 ± 927	0.444
Carbohydrates	275.8 ± 102.0	275.6 ± 105.7	0.987
Fat	113.2 ± 38.7	123.8 ± 46.6	0.182
Proteins	131.6 ± 46.6	139.8 ± 51.5	0.349
Fiber	38.2 ± 18.87	38.4 ± 20.4	0.957
Cholesterol	490.5 ± 323.6	490.5 ± 231.5	0.999
Micronutrients			
Alcohol (ethanol)	6.32 ± 11.44	4.45 ± 6.99	0.336
Calcium	1280.93 ± 532.76	1369.75 ± 650.19	0.422
Phosphorus	2036.08 ± 662.26	2103.09 ± 749.58	0.600
Total iron	23.13 ± 9.91	24.04 ± 10.12	0.597
Iodide	142.57 ± 57.56	137.29 ± 52.24	0.558
Magnesium	492.44 ± 159.05	481.81 ± 200.08	0.754
Potassium	4969.42 ± 2098.42	4928.35 ± 2112.57	0.910
Total selenium	158.24 ± 58.07	167.66 ± 72.80	0.447
Sodium	3314.16 ± 1252.21	3549.16 ± 1417.39	0.331
Zinc	13.99 ± 4.74	15.01 ± 5.82	0.302
Total niacin equivalents	84.13 ± 62.84	94.70 ± 94.59	0.509
Total folate	438.75 ± 252.76	439.99 ± 230.15	0.975
Riboflavin	3.18 ± 2.08	2.91 ± 1.29	0.229
Thiamine	1.98 ± 0.73	2.03 ± 0.82	0.727
Vitamina A, retinol equivalents of retinol and carotenoid activities	1376.16 ± 1234.01	1384.87 ± 1225.41	0.967
Vitamin B-12	11.01 ± 8.73	13.04 ± 9.31	0.203
Total B-6	4.99 ± 5.75	4.18 ± 2.61	0.404
Vitamin C (ascorbid acid)	245.58 ± 171.42	242.97 ± 179.09	0.932
**Vitamin D**	**4.84 ± 3.12**	**6.45 ± 4.49**	**0.035**
Vitamin E, alpha tocopherol equivalents of E vitamer activities	19.79 ± 9.41	20.94 ± 10.86	0.533
Food groups			
Dairy	2.50 ± 1.78	2.45 ± 1.53	0.381
Fats	1.82 ± 0.64	1.94 ± 0.65	0.374
**Meat**	**2.68 ± 1.69**	**2.73 ± 1.65**	**0.050**
**Sugars**	**1.74 ± 0.46**	**1.92 ± 0.51**	**0.056**
Legumes	2.03 ± 0.78	2.12 ± 0.79	0.542
Dried fruits	2.41 ± 0.93	2.31 ± 1.04	0.358
Cereals	2.06 ± 0.72	2.20 ± 0.64	0.242
Fish	1.70 ± 0.76	1.87 ± 0.64	0.115
Fruit	2.84 ± 1.99	2.73 ± 3.14	0.503
**Non-dairy beverages**	**2.09 ± 0.79**	**1.99 ± 0.71**	**0. 046**
Vegetables	2.44 ± 0.48	2.45 ± 0.85	0.906
Miscellany	2.26 ± 0.56	2.29 ± 0.57	0.713

Data represent mean ± sd. Differences were evaluated by an independent t test.
